# Diurnal cycle and seasonal variation of cloud cover over the Tibetan Plateau as determined from Himawari-8 new-generation geostationary satellite data

**DOI:** 10.1038/s41598-018-19431-w

**Published:** 2018-01-18

**Authors:** Huazhe Shang, Husi Letu, Takashi Y. Nakajima, Ziming Wang, Run Ma, Tianxing Wang, Yonghui Lei, Dabin Ji, Shenshen Li, Jiancheng Shi

**Affiliations:** 1 State Key Laboratory of Remote Sensing Science, Institute of Remote Sensing and Digital Earth, Chinese Academy of Sciences, Beijing, China; 2 Research and Information Center, Tokai University, Tokyo, Japan; 3 University of Chinese Academy of Sciences, Beijing, China

## Abstract

Analysis of cloud cover and its diurnal variation over the Tibetan Plateau (TP) is highly reliant on satellite data; however, the accuracy of cloud detection from both polar-orbiting and geostationary satellites over this area remains unclear. The new-generation geostationary Himawari-8 satellites provide high-resolution spatial and temporal information about clouds over the Tibetan Plateau. In this study, the cloud detection of MODIS and AHI is investigated and validated against CALIPSO measurements. For AHI and MODIS, the false alarm rate of AHI and MODIS in cloud identification over the TP was 7.51% and 1.94%, respectively, and the cloud hit rate was 73.55% and 80.15%, respectively. Using hourly cloud-cover data from the Himawari-8 satellites, we found that at the monthly scale, the diurnal cycle in cloud cover over the TP tends to increase throughout the day, with the minimum and maximum cloud fractions occurring at 10:00 a.m. and 18:00 p.m. local time. Due to the limited time resolution of polar-orbiting satellites, the underestimation of MODIS daytime average cloud cover is approximately 4.00% at the annual scale, with larger biases during the spring (5.40%) and winter (5.90%).

## Introduction

The Tibetan Plateau (TP), which is the highest and largest plateau on Earth, is well known to modulate regional and global atmospheric circulation by acting as an elevated heat source and a very large natural barrier^1–4^. The TP is more sensitive to temperature and humidity changes than the adjacent regions because of the much thinner air mass over the plateau^5,6^. The diurnal variation in cloud cover over the TP links convection and precipitation processes and has shown potential in disaster mitigation, precipitation forecasting and numerical-model validation. However, studies on these topics are scarce^7–9^, likely for the following three reasons: 1) a lack of ground observations of clouds^10^, 2) the limited temporal resolution of polar-orbiting satellites^11,12^, and 3) the inability of first-generation geostationary satellites to provide accurate cloud discrimination^13–15^. Thus, the accuracy of cloud discrimination from satellites over the TP is not fully understood.

Moderate resolution imaging spectroradiometer (MODIS) cloud cover data have been used to investigate intra-seasonal variation in cloud cover over the TP^5^ and to validate the International Satellite Cloud Climatology Project (ISCCP) cloud climatology dataset^16–18^. The MODIS instrument is carried by the Terra and Aqua satellites, which follow descending and ascending orbits and cross the Equator at approximately 10:30 and 13:30 local time, respectively^19^. Inferred cloud-cover statistics from MODIS do not consider temporal variation in clouds. The cloud dataset from the newly launched Himawari-8 geostationary satellite that includes the Advanced Himawari Imager (AHI) with 16 bands that span from 0.47 µm to 13.3 µm, facilitates the accurate determination of cloud properties with a spatial resolution of 2 km and a temporal resolution of 10 min^20^. This determination is an important advancement that can show the spatial and temporal patterns of cloud cover and be used to analyze the diurnal variation of clouds. The AHI and MODIS identify clouds with similarly high spatial resolution, wide spectral coverage and a large area over the TP; however, they provide different viewing geometries and temporal resolutions. Thus, investigating semidiurnal cycles in cloud cover over the TP with AHI and MODIS can help elucidate the uncertainties of cloud-cover statistics that are estimated from polar-orbiting satellites.

However, the estimated cloud cover from both polar-orbiting and stationary satellites is not yet sufficiently assessed^21^. The MODIS cloud mask is seldom validated over the TP, and operational AHI cloud mask is still lacking detailed accuracy assessment. The topography over the TP is complex, with large regions of mountainous areas that are covered by snow and ice that may be considered erroneously to be clouds by satellites^22,23^. In general, current cloud detection algorithms have not considered the unique topography and cloud characteristics of the TP^21^. Therefore, the accuracy of MODIS and AHI cloud detections need to be evaluated before adopting their data to investigations of cloud cover variation.

The aims of this study are to 1) validate the cloud detections from AHI and MODIS over the TP and 2) investigate deviations in monthly daytime average cloud cover as calculated from daytime twice-a-day MODIS observations and hourly AHI observations. In section 2, the dataset and analyses are described. In section 3, co-occurring AHI and MODIS estimations of cloud cover over four months are validated by using accurate cloud detections from the Cloud-Aerosol LiDAR and Infrared Pathfinder Satellite Observation (CALIPSO) instrument. Then, the semidiurnal cycles of cloud cover on a monthly scale are evaluated by using different sampling intervals from AHI measurements. We summarize our conclusions in section 4.

## Data and analytical methodology

We defined the TP region as the area from 26 to 40°N and from 73 to 105°E. The satellite datasets included the Himawari-8/AHI level-2 daily cloud mask, the Aqua MODIS Collection 6 level-2 daily cloud fractions and the CALIPSO level-2 daily vertical feature mask (VFM). The time periods considered for the AHI, MODIS and VFM data were April, July, and October of 2016 and January of 2017 to evaluate cloud detection in different seasons. Although all of the AHI, MODIS and VFM datasets include nighttime observations, only daytime data were analyzed here because the AHI nighttime cloud data had not been released at the time of writing.

The AHI cloud mask was evaluated by using a sequence of snow/ice, cloud and aerosol tests, including radiative transfer and temporal-uniformity calculations^24–27^. The AHI cloud mask was generated at a grid scale of 5 × km, and each grid was categorized into one of four categories: clear, probably clear, probably cloudy and cloudy. Both the probably cloudy and cloudy results were validated in this study. The investigation of diurnal cycles in cloud variation over the TP requires a spatial resolution of less than 7 km and a temporal resolution of less than 3 h^28,29^. In addition, the morning and evening products have incomplete coverage over the TP at solar zenith angles above 80°. Therefore, we examined only semidiurnal variations using AHI hourly cloud data that spanned 2:00–10:00 UTC.

The MODIS level-2 cloud fractions are available twice-a-day (daytime) over the TP at 10:30 and 13:30 local time from 2002 to the present at a grid scale of 5 × 5 km^30^. The cloud fraction is calculated from the level 2 cloud mask, which is determined from a large number of threshold tests and has a spatial resolution of 1 × 1 km^30^. The cloud fraction expresses the probability of cloud fraction within a grid from 0 to 100%^19^. For consistency with the AHI cloudy detections, we defined cloudy MODIS pixels as those with probabilities greater than 95%.

The VFM products provide an accurate vertical distribution of six feature types: clouds, aerosols, clear air, stratospheric features, surface, and subsurface^31,32^. LiDAR is much more sensitive to optically thin clouds than passive sensors; additionally, the cloud detection of CALIPSO is not affected by snow or ice cover^33,34^. The VFM data had a vertical and horizontal resolution of 180 m and 1667 m, respectively, at altitudes of approximately 20.2–30.1 km, a vertical and horizontal resolution of 30 m and 333 m, respectively, at altitudes of approximately 0–8.2 km, and a vertical and horizontal resolution of 60 m and 1000 m, respectively, at altitudes of 8.2–20.2 km^33^. The VFM products are based on version 3.30 (April, July, and October 2016) and 3.40 (January 2017). To match the AHI and MODIS cloud detection results, the horizontal resolution of VFM profiles were sampled to 5 km resolution from 0–30.1 km by using the center profile. In the process, only cloudy features with high- and middle- confidence were adopted; features with low confidence as well as those transparent and broken clouds (indicated by VFM cloud subtype) were discarded.

Both the CALIPSO and Aqua satellites are in the A-Train constellation, flying in close formation. Therefore, the cloud measurements from the two sensors were assumed here to be acquired at nearly the same time. CALIPSO passes the TP at approximately 7:00 UTC every day. Therefore, we obtained the AHI measurements at 7:00 UTC to correspond with the VFM results. Only the top cloud layer was considered when classifying a VFM pixel as cloudy or not. To quantitatively assess the accuracy of AHI’s and MODIS’s cloud detections, we used the false alarm rate (FAR) and Cloud Hit Rate (CHR) to evaluate performance. The FAR represents the fraction of clear pixels incorrectly classified as cloudy^22^, and the CHR represents the fraction of cloudy pixels in the dataset that are correctly classified^35^.

1 $${\rm{False}}\,{\rm{Alarm}}\,{{\rm{Rate}}}_{MODIS|AHI}=\frac{{\rm{MODIS}}|{{\rm{AHI}}}_{cloudy}\,\& \,{{\rm{VFM}}}_{non-cloudy}}{{\rm{N}}}$$

2 $${\rm{Cloud}}\,{\rm{Hit}}\,{{\rm{Rate}}}_{MODIS|AHI}=\frac{{\rm{MODIS}}|{{\rm{AHI}}}_{cloudy}\,\& \,{{\rm{VFM}}}_{cloudy}\,}{{{\rm{VFM}}}_{cloudy}}$$

where ${\rm{MODIS}}|{{\rm{AHI}}}_{cloudy}$ is the number of cloudy pixels in the MODIS or AHI cloud detections, and ${{\rm{VFM}}}_{non-cloudy}$ and VFM_*cloudy*_ are the number of non-cloudy and cloudy pixels, respectively. “&” indicates the pixels in agreement, and N is the total number of cloudy pixels detected by MODIS or AHI. According to the requirements of VIIRS cloud mask, the FAR for cloud discrimination should be within 8% in different surface types^36^. VIIRS cloud mask inherit from MODIS algorithm with many improvements. Therefore, we adopt the high-level criteria (FAR < 8%) to access the whether the performance of MODIS and VIIRS cloud discrimination is reliable over the TP.

## Results

**Figure 1:**
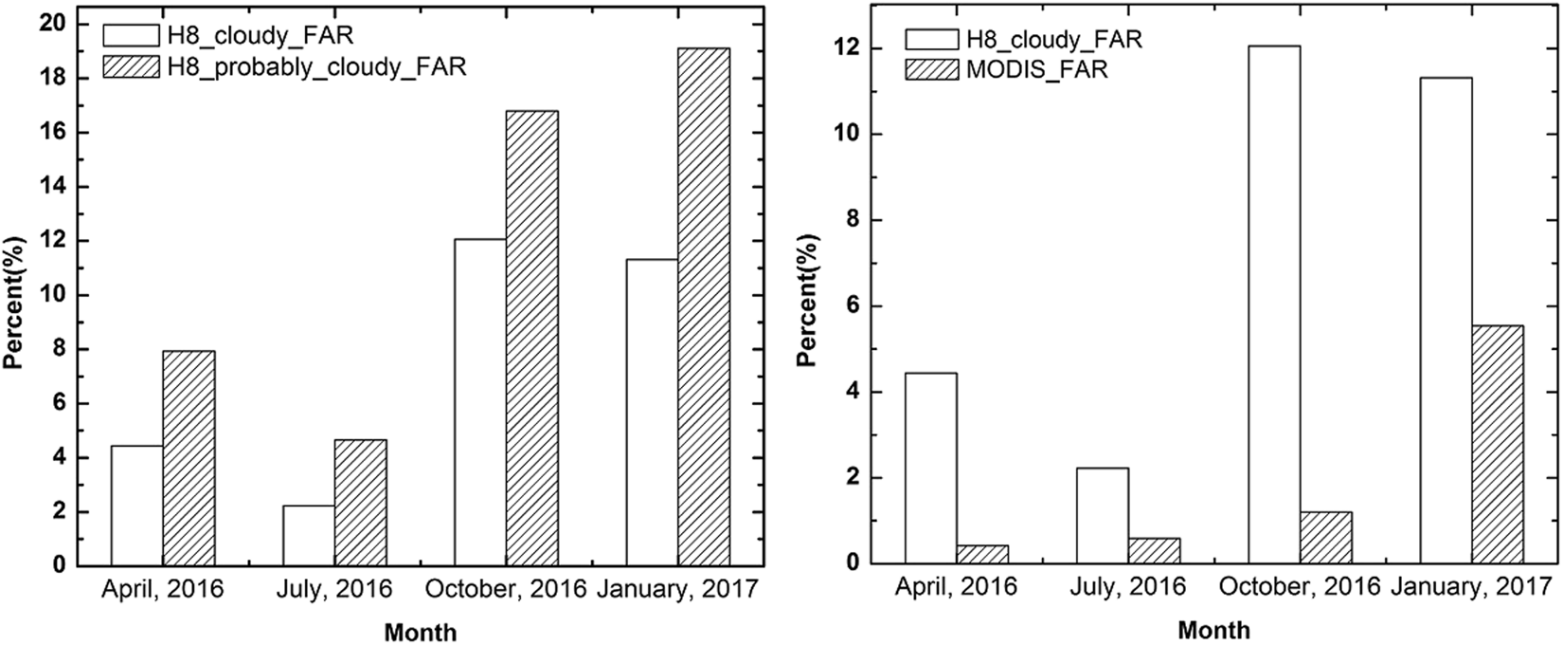
(**a**) FAR values for Himawari-8’s cloudy and probably cloudy results and (**b**) FAR values for Himawari-8 and MODIS’s cloudy results for April 2016, July 2016, October 2016 and January 2017.

Generally, higher confidence-level cloud detections indicate a higher possibility of cloud occurrence and greater cloud coverage within the grid. First, we compared AHI’s cloudy and probably cloudy pixels with simultaneous LiDAR measurements over the TP. Figure 1(a) indicates that pixels that were designated cloudy in the AHI cloud mask better matched the LiDAR observations than did the probably pixels, with an average FAR value of 7.51%. The probably cloudy pixels of AHI had an average FAR value of 12.13%. Figure 1(b) shows the joint assessment of AHI’s and MODIS’s cloudy identifications over the TP. The figure shows that the AHI cloudy results show larger bias than the MODIS cloudy results. Numbers for comparison are presented in Table 1. The average FAR values for AHI and MODIS were 7.51% and 1.94%, respectively. For the months of April, June, October, and January, the average FAR values of cloudy pixels for AHI were 4.44%, 2.23%, 12.06% and 11.32%, respectively, and those for MODIS were 0.42%, 0.59%, 1.20% and 5.54%, respectively. As shown in Table 2, the average CHR values for AHI and MODIS were 73.55% and 80.15%, respectively. For the months of April, June, October, and January, the average CHR values of cloudy pixels for AHI were 70.14%, 75.29%, 76.74% and 72.03%, respectively, and those for MODIS were 84.41%, 81.32%, 67.18% and 87.67%, respectively.

**Table 1:** False alarm rate (FAR) values for the AHI and MODIS cloud detections over the Tibetan Plateau in April, July, and October 2016 and January 2017.

Period	No. observations	AHI (%)	No. observations	MODIS (%)
April 2016	8463	4.44	8775	0.42
July 2016	9393	2.23	9393	0.59
October 2016	5330	12.06	6583	1.20
January 2017	5959	11.32	6898	5.54
Total	29145	7.51	31649	1.94

**Table 2:** Hit Rate (HR) for the AHI and MODIS cloud detections over the Tibetan Plateau in April, July, and October 2016 and January 2017.

Period	No. observations	AHI (%)	No. observations	MODIS (%)
April 2016	8463	70.14	8775	84.41
July 2016	9393	75.29	9393	81.32
October 2016	5330	76.74	6583	67.18
January 2017	5959	72.03	6898	87.67
Total	29145	73.55	31649	80.15

These FAR and CHR results indicate that MODIS’s cloudy estimates more closely matched the LiDAR measurements, partially because we implemented a very strict threshold (cloud fraction >95%) when selecting cloudy pixels for MODIS. In comparison with the MODIS cloudy results, the AHI cloudy results were generated with a relatively less strict requirement. Moreover, MODIS observes the TP at a lower orbit and from a smaller satellite viewing angles above the TP. In comparison, AHI’s orbital heights are much higher than those of MODIS, and the TP is located at the edge of the full Earth disk image of the Himawari-8 satellites; thus, it has greater geometric deformation.

**Figure 2:**
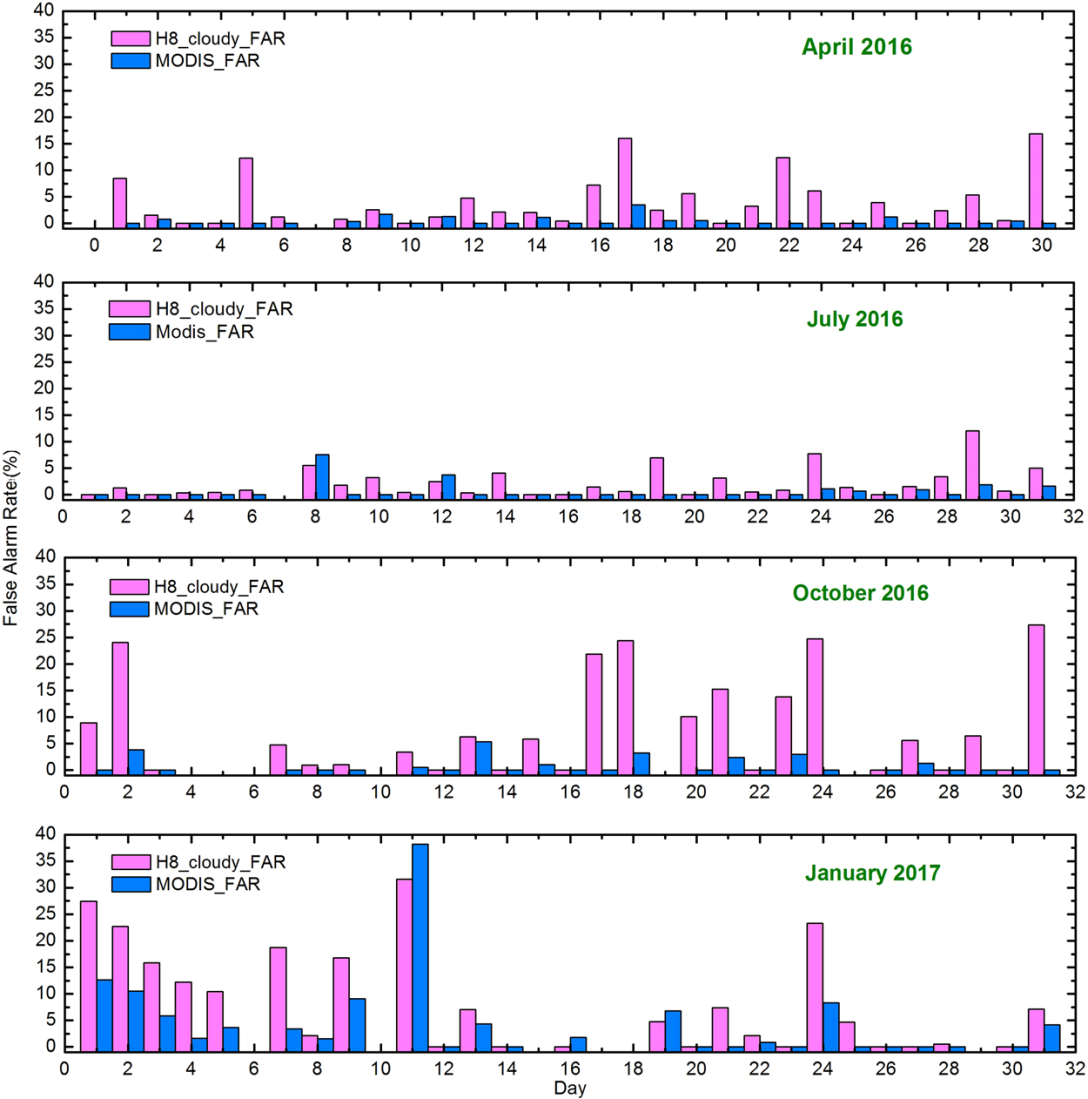
FAR values for AHI’s and MODIS’s cloud masks for April 2016, July 2016, October 2016, and January 2017; the FAR values were calculated based on the CALIPSO/VFM products.

A daily comparison of cloudy pixels between MODIS and AHI in the four investigated months was conducted to evaluate their performance in cloud detection (Fig. 2). Both AHI and MODIS yielded more accurate cloud-cover estimates during the spring and summer than during the autumn and winter. For example, the average FAR for cloudy pixels from AHI was less than 5.00% during the spring and summer, whereas the values for the autumn and winter exceeded 11.00%. Investigation of cloud-detection bias for the dates of October 31, 2016, January 1, 2017, and January 11, 2017, showed erroneous detection of snow or ice cover as clouds for both the AHI and MODIS cloud-detection algorithms. The TP is bounded by the southern and western Himalayas, the eastern Hengduan Mountains and the northern Kunlun Mountains, which have elevations that exceed 5000 m and are dominated by glaciers, and ground snow and ice in these regions increase during the autumn and winter^37,38^. We concluded that the cloudy results of MODIS show greater agreement with the VFM results than did the AHI results. In addition, although AHI and MODIS provide measurements from different platforms, both are able to discriminate clouds over the TP with a FAR of no more than 8% based on comparisons with CALIOP.

**Figure 3:**
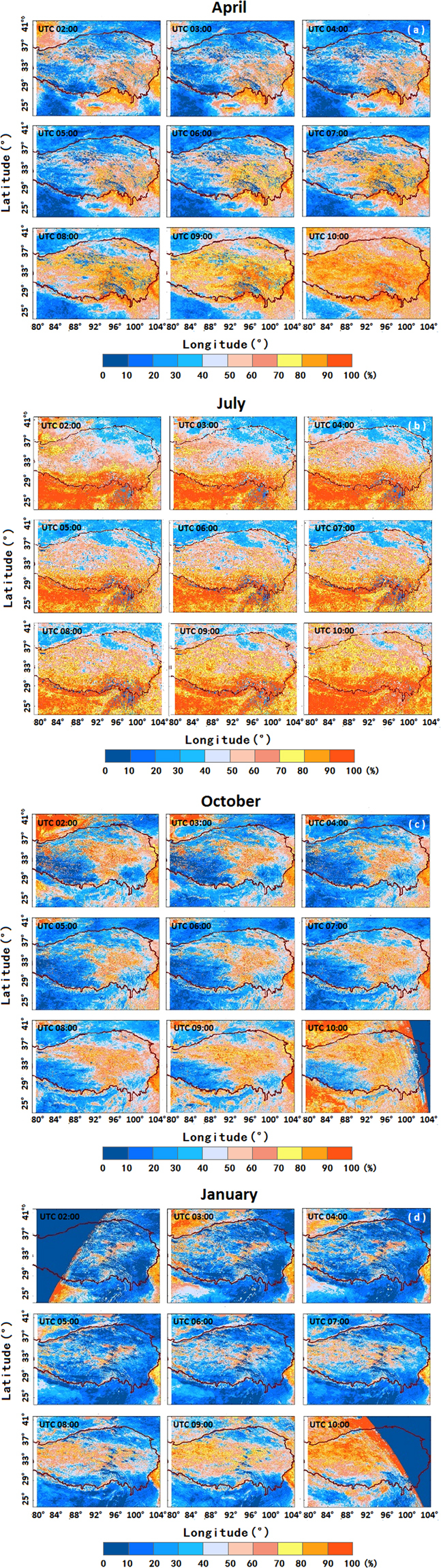
Average cloud cover from 2:00 to 10:00 UTC based on AHI data in (**a**) May, (**b**) July, (**c**) October 2016 and (**d**) January 2017. The twilight is defined with solar zenith angles of 85°~93°. Besides, the morning and evening products have incomplete coverage over the TP at solar zenith angles above 80°, therefore in October and January some regions are blank. The figures were generated using the software ArcGIS 10.1 (http://www.esrichina.com.cn).

Using hourly AHI cloudy results (UTC 2:00–10:00), a cloud cover frequency map was produced for each of April, June, October, and January (Fig. 3). Significant semidiurnal variability in cloud cover was recognized the over TP, especially in mountain areas. This phenomenon was likely caused by cumulus growth on the south-facing slopes, which were inclined towards the sun^39^. During April, the cloud fraction increased from morning to evening, especially along the eastern and southern borders of the TP. However, the semidiurnal cloud-cover cycle was weaker in July than in the other three months. July’s cloud cover over the TP was relatively high throughout the day due to the influence of monsoons^40^, especially in the southern and western-to-northern areas of the TP. Evidence of variation in cloud cover from morning to evening was apparent for April, October and January, with the mean cloud-cover frequency (MCCF) decreasing until UTC 4:00 and then increasing until UTC 10:00.

**Figure 4:**
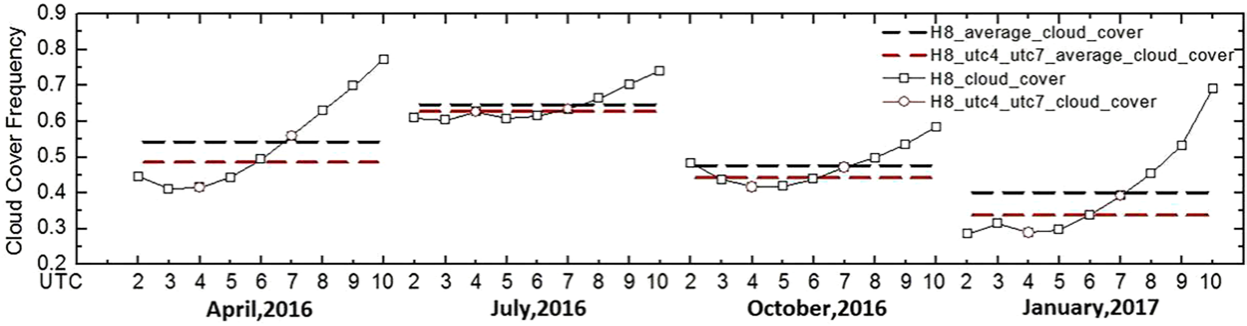
MCCF values for AHI hourly cloud cover over the TP in April, July, and October 2016 and January 2017. AHI cloud detections at UTC 4:00 and UTC 7:00 were used as Terra/MODIS and Aqua/MODIS measurements. The average FAR values for the AHI and MODIS-time measurements are displayed as black and red dashed lines, respectively.

The second aim of this study was to identify the difference in MCCF between the polar-orbiting and geostationary satellites. MCCF is the sum of all cloud-cover frequency values divided by the number of pixels and was used to express the hourly cloud occurrences for each month. AHI and MODIS acquire similar observations but at different temporal resolutions. MODIS observes the TP almost four times a day, including the daylight portions of the UTC 04 and UTC 07 orbits. We compared the MCCF values derived from twice-a-day (MODIS-time) observations with those derived from hourly AHI observations. In the calculation, we used AHI cloud detections at UTC 4:00 and UTC 7:00 as surrogates for Terra and Aqua MODIS measurements to minimize the uncertainties associated with their cloud detection results. As depicted in Fig. 4, in January, April and October, the AHI MCCF values (black line) decreased from UTC 2:00–4:00 (local time, 8:00–10:00 a.m. and then increased until UTC 10:00 (local time, 18:00 p.m.). However, in July, the values remained stable before 12:00 a.m., with few oscillations, and increased thereafter. Similar to previous studies, in the present study, upper-tropospheric cirrus clouds that originated from deep convection in mountainous areas showed maxima close to sunset^41^. Furthermore, smaller semidiurnal cycles of cloud variation occurred in July and October, 2016, because the cloud cover in these two months continued to exhibit high values.

Based on the validations, we assume that the Terra/MODIS and Aqua/MODIS observations can be represented adequately by the AHI cloud mask. We recalculated MODIS’s average cloud fraction by using the AHI cloud mask, which corresponds in time with the MODIS observations at UTC 4:00 and UTC 7:00, to examine the uncertainties that were associated with MODIS’s monthly daytime average cloud fraction without considering the semidiurnal cycle of cloud variability. Additionally, we calculated the true average cloud fraction based on the 9-h AHI cloud mask results. The black and red dashed lines in Fig. 4 represent the averaged AHI 9-h (UCT 2:00–10:00) MCCF values and averaged MODIS-time (UCT 4:00 and UTC 7:00) MCCF values, respectively. The averaged AHI MCCF values are larger than the averaged MODIS-time MCCF values in all four months. This finding indicates that if the MODIS-time cloud cover was used to calculate the monthly average daytime MCCF over the TP, the results would underestimate the MCCF because of the absence of semidiurnal cloud variation. Table 3 presents the differences between the averaged AHI 9-h and MODIS-time MCCF values. MODIS underestimated the monthly average daytime MCCF by 5.40%, 1.48%, 3.21%, and 5.90% in April, July, and October of 2016 and January of 2017, respectively. The average underestimation over these four months was 4.00%.

**Table 3:** Averaged AHI 9-h (UTC 2:00~10:00) MCCF values and averaged MODIS-time MCCF values for April, July, and October 2016 and January 2017.

Period	H8_cloud_mean (%)	H8_utc4_utc7_cloud_mean (%)	H8_minus_H8_utc4_utc7 (%)
April 2016	54.08	48.68	5.40
July 2016	64.50	63.02	1.48
October 2016	47.56	44.35	3.21
January 2017	39.91	34.01	5.90
Average	51.5125	47.515	4.00

The MODIS-time measurements are substituted by AHI cloud detections at UTC 4:00 and UTC 7:00.

The validation is limited in the following several aspects: 1) The MODIS and AHI cloud detections were validated against the VFM cloud detections. The VFM measurements are taken in orbit and cannot provide validation over large-area regions. 2) The horizontal resolution of the VFM data at three different elevations ranged from 333 m to 1667 m. We sampled the VFM data to a horizontal resolution of 5 km using only the center profile, which might have introduced bias into the resampled VFM result. 3) Multiple MODIS images are necessary to encompass the TP on any given day due to its more limited spatial coverage, which means that the cloud fields over TP are not imaged at the same time. Therefore, in the analysis of the MODIS-estimated and AHI-estimated MCCF values, we used AHI cloud detections at UTC 4:00 and UTC 7:00 to substitute Terra/MODIS and Aqua/MODIS measurements, which may differ from the MODIS measurements.

### Concluding remarks

In this study, cloud discrimination over the Tibetan Plateau is analyzed by the MODIS and AHI sensors. This study investigated the accuracy of AHI and MODIS cloud detections and evaluated the semidiurnal variation of cloud cover as derived from AHI high temporal-resolution observations. Treating the CALIPSO measurements as the truth data, our analysis of AHI and MODIS cloud detections over the TP along the CALIPSO track yielded FARs of 7.51% and 1.94%, respectively, and CHRs of 73.55% and 80.15%, respectively. The results indicate that AHI and MODIS cloud detections are reliable over the TP. We also compared diurnal cycles in the total cloud coverage in each season to investigate the estimated cloud-cover frequency from polar-orbiting and geostationary satellites. Similar trends in cloud-cover distributions occurred during all seasons, with minimum and maximum cloud-cover frequencies at 4:00 and 10:00 UTC, respectively. Due to the limit in temporal resolution, MODIS-time monthly average cloud-cover data underestimated the total cloud cover by approximately 4.00% on an annual basis. This tendency towards underestimation was greatest during the spring and winter. The temporal resolution of satellite-observed cloud climatology should be considered depending on the application.

Several unresolved issues remain. First, a more comprehensive understanding of the cloud-mask result difference of MODIS and AHI is required. Resolving this problem requires investigating instrument characteristics, algorithms and orbits. Second, a time-resolution threshold above which the average daily or monthly cloud-cover can be considered reliable may be created for regional and global cloud climatology studies. Finally, analysis of cloud property differences between sensors is an ongoing concern between the operational cloud retrieval groups^42,43^, AHI cloud mask, optical and microphysical properties estimated from CLAUDIA^44^ and CAPCOM^45^ algorithms. A comprehensive validation of AHI cloud properties on near-global scales is still needed. If AHI cloud optical and microphysical properties are accurate with polar-orbiting satellites, Himawari-8 cloud retrievals may be used in analyzing the uncertainties that are associated with the limited temporal resolution of polar-orbiting instruments in cloud variables such as the optical cloud thickness and effective radii.

### Permissions

This work is licensed under a Creative Commons Attribution 4.0 International License. The images or other third-party material in this article are included in the article’s Creative Commons license, unless indicated otherwise in the credit line; if the material is not included under the Creative Commons license, users will need to obtain permission from the license holder to reproduce the material. To view a copy of this license, visit http://creativecommons.org/licenses/by/4.0/.
